# Effect of Organic and Conventional Production Methods on Fruit Yield and Nutritional Quality Parameters in Three Traditional Cretan Grape Varieties: Results from a Farm Survey

**DOI:** 10.3390/foods10020476

**Published:** 2021-02-22

**Authors:** Gultakin Hasanaliyeva, Eleni Chatzidimitrou, Juan Wang, Marcin Baranski, Nikolaos Volakakis, Panagiotis Pakos, Chris Seal, Eduardo A. S. Rosa, Emilia Markellou, Per Ole Iversen, Vanessa Vigar, Adam Willson, Bronwyn Barkla, Carlo Leifert, Leonidas Rempelos

**Affiliations:** 1 Department of Sustainable Crop and Food Protection, Faculty of Agriculture, Food and Environmental Sciences, Universita Catollica del Sacro Cuore, I-29122 Piacenza, Italy; 2 Nafferton Ecological Farming Group, School of Agriculture, Food and Rural Development, Newcastle University, Newcastle upon Tyne NE1 7RU, UK; eleni.e.chatzidimitriou@gmail.com (E.C.); j.wang28@outlook.com (J.W.); nvolakakis@gmail.com (N.V.); pakos_panagiotis@hotmail.com (P.P.); 3 Residues and Food Safety Unit, Regulated Products Assessment Department, French Agency for Food Environmental and Occupational Health and Safety, France (ANSES), 94701 Maisons-Alfort, France; 4 Human Nutrition Research Centre, Institute of Cellular Medicine, Newcastle University, Newcastle upon Tyne NE2 4HH, UK; chris.seal@ncl.ac.uk; 5 School of Agriculture and Biology, Shanghai Jiao Tong University, Shanghai 200240, China; 6 Laboratory of Neurobiology, Nencki Institute of Experimental Biology, Polish Academy of Sciences, Pasteura 3, 02-093 Warsaw, Poland; m.baranski@nencki.edu.pl; 7 Geokomi plc, Agriculture Consultancy, P.O. Box 21, Sivas-Faistos, GR 70200 Crete, Greece; 8 Centre for the Research and Technology of Agro-Environment and Biological Sciences, Universidade de Trás-os-Montes e Alto Douro (UTAD), 5001-801, Vila Real, Portugal; erosa@utad.pt; 9 Department of Phytopathology, Benaki Phytopathological Institute (BPI), 14561 Athens, Greece; e.markellou@bpi.gr; 10 Department of Nutrition, IMB, University of Oslo, Sognsvannsveien, 0372 Oslo, Norway; p.o.iversen@medisin.uio.no; 11 Department of Haematology, Oslo University Hospital, 0372 Oslo, Norway; 12 NatMed, Southern Cross University, Military Road, Lismore, NSW 2480, Australia; vanessa.vigar@scu.edu.au; 13 Southern Cross Plant Science, Southern Cross University, Military Road, Lismore, NSW 2480, Australia; adam.willson@scu.edu.au (A.W.); bronwyn.barkla@scu.edu.au (B.B.)

**Keywords:** table grapes, polyphenols, anthocyanins, antioxidant activity/capacity, Trolox Equivalent Antioxidant Capacity (TEAC), 2,2-diphenyl-1-picrylhydrazyl (DPPH), sugar content, organic, conventional, RDA

## Abstract

The antioxidants found in grapes and wine have been linked to health benefits in humans, but may be affected by agronomic parameters, grape type/variety, and processing. Here, we report results of a farm survey which investigated the effects of production system (organic vs. conventional) and grape variety on fruit yield, total antioxidant activity/capacity (TAA, assessed by the Trolox Equivalent Antioxidant Capacity (TEAC) and 2,2-diphenyl-1-picrylhydrazyl (DPPH) radical scavenging assays), and total concentrations of total phenolics (TPC) and anthocyanins (TAC) in grapes of one red (Kotsifali) and two white (Villana and/or Vidiano) traditional Cretan grape varieties. Analysis of variance (ANOVA) results showed that grape variety choice had a more substantial effect on TPC, TAA, and TAC than primary production protocols, and significant interactions were identified between production system and grape variety choice for TAA_TEAC_. Specifically, TAA_TEAC_ was significantly (57%) higher in organic than conventional Vidiano grapes, while there was no significant effect of production system on TAA_TEAC_ in Kotsifali and Villana grapes. As expected from previous studies, the TAC was substantially higher in red Kotsifali grapes. Redundancy analysis (RDA) identified grape variety as the only strong explanatory variable/driver for yield, TPC, TAA, and TAC of table grapes, and positive associations were detected between the variety Vidiano and both TPC and TAA_TEAC_. All other explanatory variables included in the RDA (including supplementary irrigation, orchard orientation, production system, soil type, vineyard age, plant density, and fertiliser inputs) explained only a small proportion of the additional variation.

## 1. Introduction

Table grapes and wine are a rich source of phytochemicals with antioxidant activity, including polyphenols and anthocyanins [1,2], and both table grape consumption and moderate wine consumption have been linked to positive effects on human health [3,4]. Polyphenols and anthocyanins are secondary metabolites/phytochemicals that determine, or are associated with, important functions/characteristics in plants including growth, pigmentation, reproduction, and flavour [5]. They are also thought to be important components of the plants’ protective mechanisms against both biotic and abiotic stress (e.g., pathogens, predators, and ultraviolet (UV) radiation) [6]. The concentrations of antioxidant compounds in plants are known to be affected by a range of physiological and environmental factors, including ripeness of the fruit, variety choice, pedo-climatic conditions, agronomic practices, disease and pest damage, fruit maturity, and length of postharvest storage [1,6,7].

Consumer perception that organic foods have a higher sensory and nutritional quality has been a major driver for the increase in demand for organic fruit and vegetables, including grapes [8,9]. Some consumers also perceive table grapes and wine made from traditional grape varieties as having superior sensory quality characteristics, and there are efforts to preserve the growing of traditional grape varieties for future exploitation of desirable agronomic (e.g., resistance/robustness), nutritional, and sensory traits [10].

Recent systematic reviews and meta-analyses reported that organic production methods result in lower yields, but higher concentrations of phytochemical/antioxidants and higher antioxidant activity in crops, and this was primarily linked to a lower and/or more balanced supply of nitrogen when organic instead of mineral N-fertilisers are used [11,12]. However, compared with other crops (e.g., cereals and vegetables) there is limited published information on the effect of organic management practices on grape yields, as well as on sensory and nutritional quality parameters in table grapes and wine [1,2,13,14].

A recent United Kingdom (UK) retail survey confirmed that there are substantial differences in phenolic concentrations and antioxidant activity/capacity (measured by the Trolox Equivalent Antioxidant Capacity/2,2-azinobis-(3-ethylbenzothiazoline)-6-sulfonic acid (TEAC/ABTS) and 2,2-diphenyl-1-picrylhydrazyl (DPPH) methods) among grape types (white vs. red vs. black), but that significant differences in nutritional quality parameters between organic and conventional table grapes could only be detected for specific white, red, and black varieties [15]. Although retail surveys provide the most accurate estimate of product quality at the point of consumption, information on pedo-climatic conditions, specific agronomic practices used (e.g., use of supplementary irrigation), and vineyard characteristics (e.g., vineyard age, spacing, and orientation) can usually not be obtained from retailers. Confounding effects of environmental and agronomic factors that may have contributed to the differences between varieties and production systems observed can, therefore, not be identified in retail surveys [15].

There are, to our knowledge, virtually no studies that compared antioxidant/phenolic levels in indigenous, traditional Mediterranean grape varieties/landraces, and the effects of organic and conventional production methods on their nutritional quality. Although the production of many traditional varieties has declined substantially over the last 50 years, they are thought to be more adapted/resistant to local abiotic (e.g., temperature and water stress) and biotic (e.g., fungal diseases) stress factors [10,16,17]. In the context of predictions for more variable and extreme weather conditions resulting from climate change, the importance of preserving and promoting the production of indigenous, traditional grape varieties (e.g., as a genetic reservoir for resistance, robustness, and resource use efficiency traits) is, therefore, increasingly recognized [10,16,17]. Robust, traditional grape genotypes with high phytochemical/antioxidant levels may be particularly suitable for the organic/low-input sector, since they would deliver “added nutritional value” in line with consumer expectations/demands [8,9,14,18].

The main objective of the farm survey-based study reported here was, therefore, to identify the effects of production system (organic vs, conventional) on the fruit yield and the nutritional composition of one red (Kotsifali) and two white (Vidiano and Villana) indigenous, traditional Cretan grape varieties [19] that are used as table grapes and for wine production. A second objective was to compare the relative importance of (a) specific agronomic practices (e.g., fertilisation and irrigation), (b) soil type, (c) orchard parameters (e.g., age of plants and orientation of plant rows), (d) grape type/variety, and (e) production system (organic vs. conventional) on grape yield and quality parameters using redundancy analyses. Grapes were also assessed for sugar content (°Brix), which is the determinant sensory quality parameter for table grapes, as well as for wine-making. Surveys were carried out in two contrasting growing seasons to estimate potential confounding effects of climatic background conditions on grape performance.

## 2. Materials and Methods

### 2.1. Grape Farm Survey Strategy

**Figure 1:**
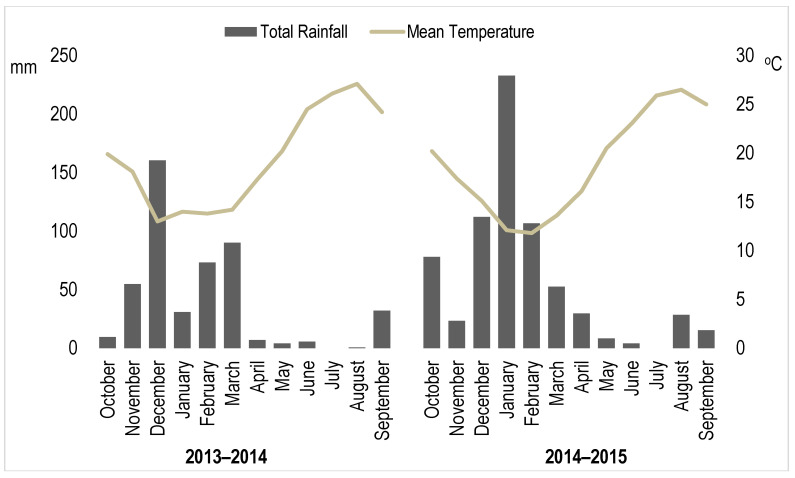
Total monthly precipitation and mean air temperature (°C) in the 2013/2014 and 2015/2016 growing seasons. Data from Knossos weather station, Heraklion prefecture, Crete, Greece (elevation: 115 m; latitude: 35°18′00″ north (N); longitude: 25°12′00″ east (E); https://stratus.meteo.noa.gr/front accessed on 1 December 2020).

The farm survey was based on collecting grape samples and soil/agronomic/vineyard data immediately before harvest on farms (vineyards) in the Heraklion region, which is a main grape production region of Crete, Greece. Grapes were collected in two successive growing seasons (2013/2014 and 2014/2015) with climatic conditions typical for the region (Figure 1). The average annual temperature was similar in both years (19.4 and 18.9 °C in the 2013/2014 and 2014/2015 season, respectively, but total annual rainfall was lower in the 2013/2014 (468 mm) than the 2014/2015 (691 mm) season. Moreover, in 2014, there was virtually no (1 mm) rain recorded in August, while, in 2015, there was substantial rainfall (28 mm) prior to harvest in August (Figure 1).

On all participating farms, data on (a) grape yields (based on the total weight of grapes harvested in fields where grape samples were taken; weights were determined at delivery to the winery) and (b) soil texture (all vineyards included in the survey had either sandy loam or clay loam soils) were recorded by investigators. A range of other vineyard and agronomic parameters were recorded via questionnaires with farmers or farm managers (see Table S1, Supplementary Materials, for further information on the farms/vineyards included in the study). The survey was repeated between mid-August and mid-September in 2014 and 2015. The three main local, organically, and conventionally grown grape varieties (Kotsifali, Vidiano, and Vilana) were chosen for the farm survey. These traditional varieties are thought to be well adapted to the local environmental and low-input agronomic background conditions for grapevine production in Crete (Dr. Manolis Kabourakis, Mediterranean University Crete, personal communication). Although mainly used for wine production, these varieties are also marketed and consumed as fresh fruit locally. Samples were collected from 22 vineyards in 2014 and 26 vineyards in 2015. From each vineyard, 10 bunches of grapes were collected randomly by walking in a zig-zag pattern through the field to generate samples covering the variation within the whole vineyard. They were placed into polyethylene cool boxes and transferred to the Livadopa experimental station (Sivas, Festos, Crete), where they were prepared for longer-term storage. Ten individual healthy grape berries were cut from each bunch using scissors. Care was taken to leave a short 0.5–1 cm stem on each grape berry, to prevent wounding-related stress responses (e.g., induction of phenolic synthesis) in the berry. One hundred berries from 10 different bunches were then placed into labelled (date, management, vineyard name, and cultivar) plastic bags and stored in a −20 °C freezer. The same procedure was repeated for a set of backup samples. All samples were transported (on dry ice) to the School of Agriculture, Food and Rural Development at Newcastle University, while the backup samples were stored in a −20 °C freezer at Geokomi Ltd. (Sivas, Crete, Greece). For sample preparation, grape berries were left to thaw for 1–2 h at ambient temperature in their plastic bags. Each bag was then emptied into an aluminium tray and weighed (about 150–200 g), followed by the removal of the short stem. Grape berries were then cut in half to allow removal of all seeds and then homogenised (only pulp and skin) for 30–120 s. Five aliquots of juice from each sample were then labelled (date, management, vineyard name, and cultivar) and transferred into a −80 °C freezer until used for different analyses.

### 2.2. Sugar and Dry Matter Content

Dry matter (DM) [20] and sugar content (SC) (OPTi Brix 54 Handheld Digital Refractometer) were determined as physical properties.

### 2.3. Chemical Reagents

Folin–Ciocalteau (FC) phenol reagent, gallic acid, potassium persulfate, and radical scavenging assay reagents (6-hydroxy-2,5,7,8-tetramethylchroman-2-carboxylic acid (Trolox), 2,2-diphenyl-1-picrylhydrazyl (DPPH), and 2,2-azinobis-(3-ethylbenzothiazoline)-6-sulfonic acid (ABTS)) were purchased from Sigma-Aldrich (St. Louis, MO, USA.). Sodium carbonate (SC), methanol, hydrochloric acid (HCl, 12 N), sodium chloride, sodium dihydrogen phosphate, sodium hydrogen phosphate, potassium chloride, sodium acetate, formic acid, acetonitrile, and methanol (MeOH HPLC grade) were supplied by Fisher Scientific (Loughborough, Leicestershire, UK).

### 2.4. Total Phenolic Content (TPC)

Total phenolic content was determined using the Folin–Ciocalteau (FC) colorimetric assay method [21]; see Hasanaliyeva et al. 2020 [15] for a detailed protocol.

Grape samples were extracted according to Tassoni, Tango, and Ferri (2013) [21]. Half a gram (0.5 g fresh weight (FW)) of homogenized grape sample was mixed with 4 mL of extraction solution (MeOH:HCl (98:2)) and incubated overnight in the dark at room temperature (i.e., in a cupboard). Extracted samples were then centrifuged at 4000 rpm (The Fisher Scientific accuSpin 3 Benchtop Centrifuge CAT No. 75004391) for 15 min at 4 °C and diluted with extraction solution (white grape samples up to 5 mL and red grape samples up to 10 mL). Grape samples were diluted with MeOH/HCl (98:2) and dilution factors of 5 and 10 were used for white and red grape samples, respectively.

Extracts from each grape sample were analysed in triplicate (technical replicates) and the mean of the three replicates was used for statistical analyses. Gallic acid (GA) solution was used as the standard for calibration curve calculations. Results were expressed as mg GA equivalent·kg^−1^ of sample fresh weight (FW).

### 2.5. Total Antioxidant Activity (TAA)

Grape samples were extracted as previously described [22] and total antioxidant activity/capacity (TAA) of the extracted samples was determined using the DPPH [23] and TEAC/ABTS [24] radical scavenging assays; see Hasanaliyeva et al. 2020 [15] for a detailed protocol. Two different assays were performed because previous studies which compared TAA in organic and conventional grapes used either the DPPH or the TEAC/ABTS assays, thus allowing results from this study to be compared with all previous comparative studies.

It should be pointed out that results obtained with the DPPH and TEAC/ABTS assays can differ [15,25]. For example, a study that compared TAA results obtained with the DPPH and ABTS/TEAC assay for a wide range of antioxidant-rich fruits, vegetables, and beverages with published oxygen radical absorbance capacity (ORAC) data from the USDA database reported that “antioxidant capacity detected by ABTS was significantly higher compared to that by DPPH” and concluded that (a) “high-pigmented and hydrophilic antioxidants are better reflected by ABTS assay than DPPH assay” and (b) the “ABTS assay may be more useful than DPPH assay for detecting antioxidants in a variety of foods” [25].

Undiluted extracts from each grape sample were analysed in triplicate (technical replicates), and the mean of the three replicates was used for statistical analyses.

### 2.6. Total Anthocyanin Content (TAC)

Grape samples were extracted as previously described [15], and total anthocyanin content (TAC) was measured using the pH differential method with slight modifications as described previously [15,26].

Grape samples were diluted with pH buffers, and dilution factors of 5 and 10 were used for white and red grape samples, respectively. Each diluted grape sample was analysed in triplicate (technical replicates), and the mean of the three replicates was used for statistical analyses.

The diluted grape samples were then used for spectrophotometric analyses (using a UV–visible light (Vis) spectrophotometer (Shimadzu UV mini-1240, Shimadzu UK Ltd, Milton Keynes, Buckinghamshire, UK)), and absorbance was assessed at 520 nm (A_520_) and 700nm (A_700_). Final absorbance was calculated according to the formula A = (A_520nm_ − A_700nm_) pH 1.0 − (A_520nm_ − A_700nm_) pH 4.5. Two of the most common anthocyanin pigments (cyanidin-3-*O*-glucoside and malvidin-3-*O*-glucoside) were used as equivalents in the calculation. Results were expressed as mg·kg^−1^ of sample fresh weight (FW) (for grape samples) or mg·L^−1^ (for wine samples).

### 2.7. Identification and Quantification of Individual Anthocyanins by High Performance Liquid Chromatography (HPLC)

Concentrations of individual anthocyanins in grape samples were detected and quantified according to Kammerer, Claus, Carle, and Schieber (2004) [27] with slight modifications. Aliquots of 0.5 g of grape juice sample were mixed with 1.5 mL of 0.1% acidified methanol and vortexed for 2 h for complete extraction. Samples were then centrifuged at 10,600 rpm (Fisherbrand™ accuSpin™ Micro 17/Micro 17R Microcentrifuges; Fisher Scientific, Loughborough, Leicestershire, UK) for 5 min, and the supernatant was transferred into a second tube. The extraction was repeated adding 0.5 mL of 0.1% acidified methanol into the remaining residue and vortexed for another 15 min. Extracts were then centrifuged, and the supernatants were combined and re-centrifuged. After centrifugation, extracts were passed through a 0.45 μS, 25 mm filter (Dutscher Scientific UK Ltd., Syringe Filter Nylon, non-sterile) and stored at −80 °C until analysis by HPLC.

Analyses and separation of individual anthocyanin components were performed using a Phenomenex, Synergi^TM^ 4 μm Hydro-RP 80 Å (C18 phase, 250 × 4.6 mm) column, fitted with a C18 guard column (3.2–8.0 mm internal diameter (ID)) at a temperature of 25 °C. The Shimadzu HPLC system (Shimadzu UK Ltd, Milton Keynes, Buckinghamshire, UK) was equipped with LabSolution software, a DGU-20A3R degasser, two LC-20AD pumps, an SIL-20AC HT autosampler, an SPD-M20A diode array detector, and a CTO-20AC column oven. The detector was set to an acquisition range of 190–700 nm.

Water/formic acid/acetonitrile (A) (87:10:3) and water/formic acid/acetonitrile (B) (40:10:50) were used as the mobile phase with a flow rate of 0.8 mL·min^−1^. The gradient programme for the mobile phases (A:B) was as follows: 0.02 min (10:90), 5 min (10:90), 15 min (25:75), 20 min (31:69), 25 min (40:60), 35 min (50:50), 45 min (100:0), 50 min (10:90), and 55 min (10:90). An injection volume of 50 μL was used for all samples, and quantification was performed at 520 nm.

Identification was based on peak relative retention times and elution order of chromatograms obtained by Kammerer et al. (2004) [27]. Individual anthocyanins were quantified using a calibration curve of malvidin-3-*O*-glucoside in the range of 50 to 0.05 µg·mL^−1^.

### 2.8. Identification of Individual Anthocyanins by LC–MS

LC–MS analyses to confirm the identity of anthocyanins identified by HPLC analysis was carried out by the Newcastle University Protein and Proteome Analysis (NUPPA) laboratory based on previously described methods [27,28,29]. Anthocyanin extracts were provided in a neat and 1/10 dilution. Samples were acidified with trifluoroacetic acid (TFA) to a final concentration of 0.1% (*v*/*v*). Each sample was analysed with an individual LC–MS experiment using a Thermo RSLC Nano LC (www.thermofisher.com accessed on 1 December 2020, Gloucester, UK) coupled to a Sciex 6600 mass spectrometer (www.sciex.com, Framingham, MA, USA) Mobile phases were made as follows; loading buffer 4% (*v*/*v*) acetonitrile with 0.1% (*v*/*v*) TFA, buffer A (4% acetonitrile 0.1% formic acid (FA)), and buffer B (80% acetonitrile 0.1% FA). Separation was carried out using a linear gradient from 4–80% Buffer B over 40 min. This was followed by a 10 min wash at 90% Buffer B, and then a column equilibration at 4% Buffer B to return the column to original starting conditions. Next, 5 μL samples (1/10 dilution) were loaded onto the 300 μm C18 trap column for desalting before being resolved on a 23 cm 75 μm ID home-packed analytical column containing Dr Maisch 3 μm particle size stationary phase. Analytes were injected online into the mass spectrometer, which acquired data in a data-dependant format. Survey scans were performed over an *m*/*z* range of 400–1200. From each survey, the 30 most intense ions were selected for MS/MS; charge states +1 to +5 were considered for MS/MS. Precursors were fragmented with a rolling collision energy, as a function of the charge state of the peptide ion. The total cycle time was 1.7 s.

Data were visualised using Analyst v2.2 (Sciex). Extracted ion chromatograms, *m*/*z* anthocyanin values, and respective MS/MS spectra for relevant *m*/*z* were exported and compared with previously published data [27,28,29].

### 2.9. Statistical Analysis

The effects and interactions between factors on measured parameters were assessed by analysis of variance (ANOVA) derived from linear mixed-effects (LME) models [30] by using the *nlme* package in R [31]. The hierarchical nature of the design was reflected in the random error structures that were specified as farm/year/management/variety. The normality of the residuals of all models was tested using Quantile-Quantile (QQ) plots. Differences between the grape varieties or the interactions between factors were tested using Tukey contrasts in the general linear hypothesis testing (GLHT) function of the *multcomp* package in R. A linear mixed-effects model was used for the Tukey contrasts, containing a treatment main effect, with three levels, with the random error term specified as described above. The relationships between soil, orchard, and agronomic factors (recorded on participating farms via structured questionnaires) and table grape yield and quality parameters were investigated by redundancy analysis (RDA) using the CANOCO 5 software [32]. Automatic forward selection of the variety, environmental, and agronomic factors was used within the RDAs, while their significance in explaining additional variance was calculated by using Monte Carlo permutation tests. The amount of total N, P, and K with organic fertilisers was estimated on the basis of average published N, P, and K contents of the different organic fertilisers used (Table S1, Supplementary Materials).

Due to the small number of yield and quality response variables available for RDA, the number of explanatory variables/drivers was restricted to nine. In the biplot derived from RDA shown in Figure 1, production systems, variety, soil type, irrigation, vineyard age, plant density, and estimated total nitrogen (N) and potassium (K) inputs were used as explanatory variables/drivers. In the biplot resulting from RDA shown in Figure S1 (Supplementary Materials), we used variety, soil types, orchard orientation, irrigation, vineyard age, plant density, and estimated total N, P, and K inputs as explanatory variables/drivers.

## 3. Results

In the farm survey reported here, grapes of two white varieties (Villana and Vidiano) and one red variety (Kotsifali) were collected from 13 organic and 13 conventional grape orchards (Table S1, Supplementary Materials) in two consecutive years. Red wines made from Kotsifali and white wines made from Vidiano grapes were also collected from wineries that were supplied by farms included in the grape survey. Table grapes and wines were assessed for (a) dry matter (DM) and sugar content (° Brix), (b) total phenolic content (TPC), (c) total antioxidant activity (TAA) assessed by two different methods TAA_DPPH_ and TAA_TEAC_, (d) total anthocyanin concentration (TAC) assessed by two different equivalences (TAC_cyan_ and TAC_mal_), and (e) anthocyanin profiles (Table 1 and Table 2).

### 3.1. Yields, Dry Matter and Sugar Content in Grapes

The dry matter content and sugar levels in grape pulp and grape juice were slightly, but significantly higher (by 8%, 16%, and 13%, respectively) in 2014 than 2015, but there was no significant effect of year on grape yields. There were no significant effects of variety and production system on grape yields, dry matter, and sugar content detected (Table 1). However, we observed substantial variation in grape yield between orchards (especially for Villana); numerically, Villana had the highest average yields (15.7 t·ha^−1^), followed by Kotsifali (14.6 t·ha^−1^) and Vidiano (12.5 t·ha^−1^) (Table 1).

### 3.2. Antioxidant Activity and Phenolic and Anthocyanin Content in Grapes

The TPC, TAA_DPPH_, and TAA_TEAC_ values were significantly higher (by 43%, 12%, and 96%, respectively) in the 2013/2014 season (which had no rainfall during the harvest period; Figure 1) than the 2014/2015 season (which had significant rainfall prior to harvest in August; Figure 1), and there were also trends (0.01 > *p* > 0.05) toward significantly higher TAC_cyan_ and TAC_mal_ in 2013/2014 (Table 1). However, for TAA_DPPH_, TAC_cyan_, and TAC_mal_, significant interactions between year and variety were also detected. When these interactions were examined further, concentrations of TAA_DPPH_ and TAC were found to be significantly higher in 2014 than 2015 in Kotsifali grapes only (Table S2, Supplementary Materials). No significant effects of year on the profiles of individual anthocyanins in the red grapes (Kotsifali) could be detected (Table S3, Supplementary Materials).

A significant main effect of production system was only identified for the TAA_TEAC_, which was 16% higher in organic compared with conventional grapes (Table 1). However, for TAA_TEAC_, there was also a significant interaction between production system and grape variety (Table 1). When this interaction was further examined, organic Vidiano grapes were found to have significantly (57%) higher TAA_TEAC_ than conventional Vidiano grapes, while organic and conventional grapes of the varieties Kotsifali and Villana had similar TAA_TEAC_ (Table 2).

Anthocyanin profiles were only analysed in red grapes (Kotsifali), and no significant effect of production system could be detected (Table S3, Supplementary Materials).

Significant main effects of variety were detected for TPC, TAA, and TAC (Table 1). As expected from previous studies (see Section 4), only red grapes contained substantial concentrations anthocyanins, while white grapes contained virtually no anthocyanins (Table 1). The finding that TPC and TAA_TEAC_ were significantly (~60% and ~85%, respectively) higher in red Kotsifali than white Villana grapes was also as expected from previous studies (Table 1).

However, in this study, the traditional white variety (Vidiano) overall had similar TPC and TAA_TEAC_ when compared with the red variety (Kotsifali). This and the finding that organic white Vidiano grapes had similar TAA_TEAC_ when compared to organic red Kotsifali grapes, while conventional Vidiano grapes had significantly lower TAA_TEAC_ than conventional Kotsifali grapes, were unexpected (Table 2). It should be pointed out that TAA_DPPH_ was significantly (~95%) higher in Kotsifali than both Vidiano and Villana grapes, and that there was no significant difference in TAA_DPPH_ between Vidiano and Villana (Table 1).

### 3.3. Associations between Variety and Agronomic Drivers and Grape Yield and Composition

**Figure 2:**
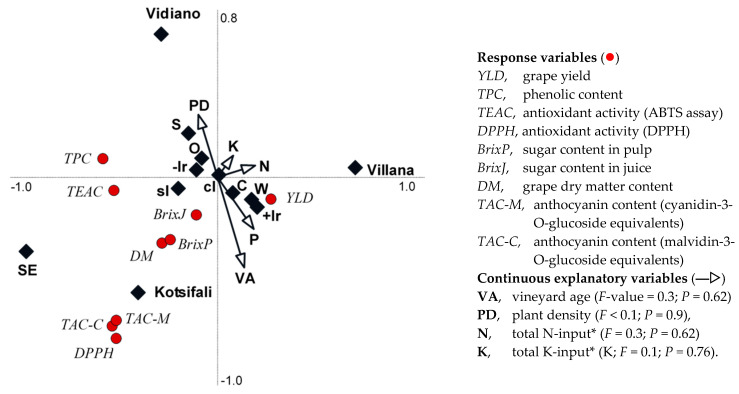
Biplot derived from the redundancy analysis showing the relationship between variety, production system, agronomic, and orchard site and soil explanatory variables/drivers and grape yield and quality parameters. Eigenvalues were 32.5% and 8.6% for Axis 1 and 2, respectively. Fixed explanatory variables (◆) were (a) variety: **Vil**, Villana (*F* = 17.1, *P* = 0.002); **Vid**, Vidiano (*F* = 5.7, *P* = 0.018); **Kot**, Kotsifali (*F* = 5.7; *P* = 0.018), (b) irrigation: **+Ir**, with drip irrigation (*F* = 1.3, *P* = 0.27); −**Ir**, without drip irrigation (*F* = 1.3, *P* = 0.27), (c) orchard orientation, facing: **W**, west (*F* = 0.9, *P* = 0.36); **S**, south (*F* = 0.6, *P* = 0.44); **SE**, southeast (*F* = 0.6, *P* = 0.44), (d) production system (organic (**O**), *F* = 0.6, *P* = 0.44; conventional (**C)** F = 0.6; *P* = 0.44), and (e) soil texture: **cl,** clay loam (*F* = 0.4, *P* = 0.59); **sl**, sandy loam (*F* = 0.4, *P* = 0.59). * From mineral and/or organic fertilizer.

Similar to the ANOVA, the RDA also identified grape type/variety as the strongest (*F*_Villana_ = 17.1; *F*_Vidiano_ = 5.7; *F*_Kotsifali_ = 5.7) and only significant (*P*_Villana_ = 0.002; *P*_Vidiano_ = 0.018; *P*_Kotsifali_ = 0.018) explanatory variable/driver for the grape composition parameters assessed (Figure 2). The strength (*F*-value) of all other explanatory variables included in the RDA was substantially lower and decreased in the following order: irrigation (*F*_with irrigation_ = 1.3; *F*_without irrigation_ = 1.3) > orchard orientation (*F*_south-east facing_ = 0.6; *F*_south facing_ = 0.6; *F*_west facing_ = 0.9) > production system (*F*_organic_ = 0.6; *F*_conventional_ = 0.6) > soil type (*F*_clay loam_ = 0.4; *F*_sandy loam_ = 0.4), while vineyard age, plant density, and estimated total nitrogen and potassium inputs explained very little (*F* ≤ 0.2) of the additional variation (Figure 2).

The RDA drivers explained approximately 41% of the variation (32.5% by axis 1 and a further 8.6% by axis 2) associated with the grape composition response variables assessed (Figure 2). There were strong positive associations between the red grape variety Kotsifali and total anthocyanin content and TAA_DPPH_, as well as a weaker positive association with TAA_TEAC_, DM, and sugar content along both the negative axes 1 and 2. There was also a positive association between the variety Vidiano and TPC (along both the negative axis 1 and positive axis 2) and TAA_TEAC_ along the negative axis 1. In contrast, for the variety Villana, RDA identified negative associations with all composition parameters assessed, but a positive association with grape yield along the positive axis 1 (Figure 2).

There were also weak positive associations between grape yield and the use of supplementary irrigation, west-facing orchards, higher N and K inputs, clay soils, older orchards, and conventional crop management along the positive axis 1 (Figure 2). In contrast, there were weak positive associations between all nutritional composition parameters and higher planting density, southeast- and south-facing orchards, sandy loam soils, and non-use of irrigation along the negative axis 1 (Figure 2). When production system was replaced with total estimated P-input in the list of explanatory variables used for RDA, very similar results were obtained (Figure S1, Supplementary Materials).

## 4. Discussion

A range of previous studies analysed the effects of pedo-climatic conditions, agronomic protocols, grape type/variety, region, and/or vintage on sensory and nutritional quality parameters in table grape and/or wine [15,21,33,34]. A number of studies assessed the effects of organic production methods on grape quality parameters, but reported variable trends (Table 3, Table 4 and Table 5).

However, there is very limited information on the performance of indigenous, traditional grape varieties in organic and conventional production systems. This is, to our knowledge, the first study in which a significant interaction between production systems (organic vs. conventional) and different traditional Cretan grape varieties is reported. The study was based on farms in a traditional Cretan wine-growing region with a very uniform temperature and rainfall pattern, to minimise potential confounding effects of variable environmental background conditions. We recorded a range of orchard (e.g., soil type, age, and orientation) and agronomic (e.g., irrigation and NPK inputs with fertilisers) parameters on all farms. This allowed us to investigate possible associations between grape yield and quality parameters and (a) grape variety, (b) production system (organic vs. conventional), and (c) specific agronomic and orchard/site explanatory variables/drivers by RDA.

### 4.1. Effect of Variety and Production System on Grape Yields

The finding that grape yields of all three varieties were similar in organic and conventional production was unexpected, since a meta-analysis of comparative studies reported that crop yields are higher in conventional compared with organic production [12].

The absence of significant yield differences between organic and conventional production may have been due to low downy mildew disease severity. This is the most important factor affecting grape yields and quality and is more difficult to control in organic farming systems, where the use of synthetic chemical fungicides is prohibited and farmers are only allowed to use relatively inefficient Cu-fungicides [15,16]. Crete has a semiarid climate (there is virtually no rainfall and low relative humidity between June and August/early September when grapes are harvested), which is known to result in much lower downy mildew disease pressure and associated crop losses than in many other European grape-growing regions [15,16]. However, since the varieties included in this study are primarily used for wine-making, the lack of yield differences between organic and conventional production systems may also have been due to lower fertiliser (in particular N) inputs in grapes primarily used for wine-making [35].

Similarly, the finding of no significant differences in yield between varieties was unexpected, but may also have been due to the relatively low-input (especially with respect to fertiliser and supplementary irrigation) agronomic protocols used in both organic and conventional production in Crete and/or the large variability in yields between orchards observed for the variety Villana, which numerically produced the highest yields.

This view is supported by results from RDA, which indicated that (a) variety choice was the strongest explanatory variable/driver for both yield and grape quality parameters, and (b) there were trade-offs between yield and nutritionally relevant antioxidant concentrations and activity. These RDA results are consistent with previous studies, which reported that the intensification (e.g., increased use of irrigation, fertiliser inputs, and/or crop protection products) of agronomic practices to increase yields may cause a “dilution effect” resulting a reduction in antioxidant/phytochemical and mineral micro-nutrient concentrations in crops [11,35,36]. In this study, explanatory variable/drivers that were positively associated with increased yield (e.g., the variety Villana and, to a lesser extent, the use of drip irrigation, higher N and K inputs, clay soils, conventional production systems, west-facing orchards, and older plants) were also negatively associated with antioxidant concentrations, activity, sugar, dry matter, and anthocyanin and phenolic concentrations.

### 4.2. Effect of Year, Variety, and Production System on Table Grape Quality Parameters

The finding of higher phenolic and antioxidant activity in the 2013/2014 season compared with the 2014/2015 season was expected and most likely due to farmers harvesting grapes before the optimum ripening stage in 2015 to avoid fungal infection/spoilage of grapes after the unusually high rainfall in late August (Figure 1). This view is supported by the lower sugar content found in grapes harvested in 2015 compared with those harvested in 2014, since sugar content was shown to increase in grapes over time [2,6]. Wineries in the Heraklion area recommend that grapes of all three varieties are harvested early in years with heavy rainfall in late August/early September (personal communication, Dr Manolis Kabourakis, Hellenic Mediterranean University, Crete, Greece).

Differences between production years were also reported in a recent table grape retail survey [15].

Results reported here show that overall variety choice had a substantially larger effect on antioxidant activity and concentrations than either production system (organic vs. conventional) or non-production system-specific agronomic parameters such as the use of supplementary irrigation and total N, P, and K inputs and orchard parameters (soil type, age, orchard orientation, and plant density). However, the study also demonstrated that production system can have substantial effects on grape quality in specific varieties (higher TAA_TEAC_ activity in organic than conventional Vidiano grapes). These results are consistent with previous studies that compared antioxidant activity and phenolic concentrations in different grape types (white, red, and black) and organic and conventional grapes of the same variety (see studies listed in Table 3, Table 4 and Table 5). Furthermore, as previously reported for other white grape varieties [15], the varieties Vidiano and Villiana were found to contain virtually no anthocyanins.

Previous studies also reported higher antioxidant activity and/or concentrations in red/black than white grapes and differences in concentrations between varieties belonging to the same grape type (white or black/red) (Table 3, Table 4 and Table 5). Moreover, for many varieties included in previous comparative studies, no significant composition differences between organic and conventional grapes were found, and there was no consistent effects of production systems for those varieties in which significant differences in TAA, TPC, and/or TAC between organic and conventional grapes were detected (Table 3, Table 4 and Table 5). For example, a study by Tassoni et al. (2013, 2014) [21,34] reported significantly higher TAA_TEAC_ in organic white grapes of the variety Albana and organic red grapes of the variety Lambrusco, but also in conventional red grapes of the variety Sangioves, while production system had no significant effect on TAA_TEAC_ in white grapes of the variety Pignoletto (Table 3, Table 4 and Table 5). Similarly, a recent retail survey [15] reported significantly higher TPC in organic table grapes of the white variety Sugarlone, but also in conventional table grapes for the white variety Prime, while there was no significant effect of production system for the white varieties Early Sweet and Thompson (Table 3).

The reasons for the variable and sometimes contrasting effects of production systems on antioxidant activity, TPC, and TAC are poorly understood. Results from long-term factorial field experiments with arable crops have suggested that higher nitrogen supply/availability from mineral N-fertilisers results in lower phenolic and flavonoid concentrations in conventional wheat grain and found no evidence for pesticides (herbicides, fungicides, and growth regulators) used in conventional farming contributing to the differences in antioxidant levels between organic and conventional wheat grain [36]. However, increased expression of phenolic compounds and other secondary plant metabolites with antioxidant activity in plants may also be induced by biotic (pest and disease attack) and abiotic stress factors (e.g., drought, flooding, or heat stress) [36,39,41]. Differences in pest and disease pressure and/or the relative efficacy of crop protection methods used in organic and conventional grape production may, therefore, also contributed to the variability of results reported in different studies (Table 3, Table 4 and Table 5).

There were differences in climatic conditions (e.g., total precipitation and amount of rainfall prior to harvest) between the two growing seasons in which surveys were carried out. The finding of differences in dry matter, sugar content, TPC, TAA_TEAC_, and TAA_DPPH_ between growing season/years was, therefore, not surprising. This is consistent with the results of previous studies that identified not only variety but also geographical location and climatic conditions as important drivers for antioxidant activity and/or concentrations in grapes [15,42,43]. It is, therefore, likely that the variable effects of production system reported here and in previous studies (Table 4) were due to complex interactions among climatic conditions, variety choice, and production methods, as previously shown for cereals [36]. However, long-term controlled factorial experiments with grapes would be required to identify and quantify such interactions.

The production of indigenous, traditional grape varieties in Crete (and other regions of the Mediterranean) declined significantly between the 1960s and early 2000s, but has increased again more recently [18]. The reasons for this resurgence are largely unknown, but an agronomist in Crete (Dr Manolis Kabourakis; personal communication) and the farmers included in the survey described that this was mainly due to the ability to market indigenous varieties as table grapes and for wine-making, leading to increasing demand for table grapes and wine made from traditional varieties from both locals and tourists, as well as the greater robustness/resistance of traditional varieties in low-input and organic production systems.

White grapes are well known to have significantly higher phenolic and antioxidant concentrations than red and black grapes [15] (Table 3, Table 4 and Table 5). The finding that one traditional white variety (Vidiano) produced similar total phenolic (TPC) and antioxidant activity (TAA_TEAC_) levels to red grapes of the variety Kotsifali was, therefore, unexpected. Furthermore, both traditional Cretan white grape varieties had relatively high TPC and TAA levels compared with other white grape varieties (e.g., those included in previous studies that compared the nutritional composition of organic and conventional grapes; Table 3). This may be significant in terms of increasing the use of traditional white grape varieties by farmers/producers since high antioxidant/phenolic concentrations were linked to disease resistance traits in plants and may facilitate the development of marketing strategies focused on “added nutritional value” to increase consumer demand [11,36].

By minimising confounding effects of variable environmental conditions in this study (the farms included in the survey were all in a region with very similar climatic conditions), we were also able to confirm results of a recent retail survey which suggested that the nutritional composition of grapes is determined by interactions between variety choice and production system [15] (Table 3, Table 4 and Table 5). Specifically, in this study, organic white Vidiano grapes had a higher TAA_TEAC_ content than both organic white Villana and organic red Kotsifali grapes, while both conventional Vidiano and conventional Villana grapes had a significantly lower TAA_TEAC_ content than conventional Kotsifali grapes.

White grapes are known to contain anthocyanins but at very low levels [45,46] and are, therefore, often thought to have a lower nutritional value than red and black grapes [15]. The finding that organically produced Vidiano grapes had similar or higher antioxidant activity (TAA_TEAC_) and/or total phenolic levels when compared to many red/black grape varieties examined in this and previous studies [1,2,7,13,15,21,34,44] is, therefore, important. Specifically, it demonstrates, for the first time, that it is possible to identify traditional white varieties that compensate for low anthocyanin content with higher expression of other phenolics, thus providing similar levels of nutritionally desirable phenolics and antioxidant activity.

Evidence from a recent retail survey [15] and the farm survey reported here, therefore, indicates that selection of grape varieties adapted to producing higher TPC and TAA contents under specific production environments may be a suitable strategy to improve the nutritional quality in both organic and conventional grape products.

It should be pointed out that, although most previous studies reported in Table 3, Table 4 and Table 5 used similar analytical methods, even small differences in sample preparation and analytical protocols may have affected the results obtained for TPC, TAA, and TAC measurements [1,2,7,13,15,21,34,44]. Differences among varieties should, therefore, be confirmed in future studies which use identical protocols for all varieties studied.

### 4.3. Potential Nutritional Impacts of Contrasting Antioxidant Levels in Grapes

There is increasing evidence that the antioxidant (mostly (poly)phenolic) compounds in food crops have protective effects against a range of chronic diseases, but there is still limited information on the exact physiological mechanism underlying these effects [1,3,4]. Consequently, whether these compounds act in the gastrointestinal environment, as “antioxidants” once metabolised in the intestine and absorbed, and/or as signalling molecules requires further investigation.

However, several epidemiological studies have linked the consumption of grapes and grape products to a reduced risk of various chronic diseases including cardiovascular diseases, some cancers, and neurodegenerative diseases [3,4]. Selecting table grape varieties and wines with high antioxidant, TPC, and TAC levels may, therefore, deliver additional benefits, since this would allow an increased intake of nutritionally desirable phytochemicals without an increase in calorie and/or alcohol consumption.

The finding that antioxidant activity and concentrations were broadly similar in organic and conventional grapes samples may suggest that there is no nutritional benefit of switching to organic table grape consumption. However, to what extent organic table grape consumption may provide health benefits remains unclear from the results reported here. This is mainly because other nutritionally relevant compounds that are known to be affected by production system (e.g., mineral micronutrient, cadmium, and pesticide residue levels) were not assessed in this study, and there is limited information in the existing literature on differences in mineral, toxic metal, and pesticide residues between organic and conventional grapes [7,11].

### 4.4. Limitations of the Study

The main limitation of the study reported here was the inability to obtain detailed information on (a) crop protection protocols and (b) certain non-production system-specific management parameters (e.g., amount of irrigation water used, tillage, and ripening stage of grapes at harvest), (c) foliar disease severity, and (d) processing methods and storage conditions used for wines included in the survey. More detailed recording of these factors in farm surveys and additional assessment of mineral nutrients, toxic metal, and pesticide residues should be considered for future comparative studies of yield and nutritional and sensory quality difference in organic and conventional table grapes.

## 5. Conclusions

Results reported here suggest that, in regions with relatively extensive grapevine production systems, organic and conventional agronomic protocols produce broadly similar yields and nutritional composition when traditional, local varieties are used. However, the finding that organic grapes of one white grape variety (Vidiano) had higher antioxidant activity than its conventional comparators suggests that production system can have an effect on the nutritional composition for certain varieties.

Red and black grapes are often considered to have a higher nutritional value than white grapes due to their higher anthocyanin content. The finding that organically produced Vidiano grapes had similar antioxidant activity and higher phenolic levels than the grapes of the red variety Kotsifali demonstrates the potential for identifying traditional white varieties which compensate for the low anthocyanin content with higher expression of other phenolics.

## Figures and Tables

**Table 1:** Effect of, and interaction among, production system (organic (ORG) vs. conventional (CONV)), variety, and year for the yield, dry matter content (DM), sugar content (SC) of pulp/juice, total phenolic content (TPC), total antioxidant activity (TAA) using the 2,2-diphenyl-1-picrylhydrazyl (DPPH) or Trolox Equivalent Antioxidant Capacity (TEAC (assays and total anthocyanin content (TAC) (expressed as cyanidin-3-*O*-glucoside (cyan) and malvidin-3-*O*-glucoside (mal) equivalents) in table grapes of the Cretan local varieties Kotsifali, Villana, and Vidiano (three-factor ANOVA). TE, Trolox equivalent.

Factors	Yield (t/ha)	DM (%)	SC Pulp (°Brix)	SC Juice (°Brix)	TPC (mg GAE·kg^−1^)	TAA (DPPH) (µmol TE·g^−1^)	TAA (TEAC) (µmol TE·g^−1^)	TAC (mg cyan·kg^−1^)	TAC (mg mal·kg^−1^)
**Year (Yr)**									
2014 (*n* = 22)	14.6 ± 1.1	23.0 ± 0.6	21.5 ± 0.7	21.5 ± 0.5	2037 ± 150	84 ± 6	14.3 ± 1.1	432 ± 47	456 ± 50
2015 (*n* = 26)	14.2 ± 1.1	21.3 ± 0.5	18.5 ± 0.4	19.1 ± 0.5	1423 ± 94	75 ± 5	7.3 ± 0.6	296 ± 23	313 ± 25
**Production** **System (PS)**									
ORG (*n* = 24)	14.0 ± 1.3	21.6 ± 0.6	19.2 ± 0.5	19.7 ± 0.6	1770 ± 139	79 ± 6	11.3 ± 1.2	341 ± 48	360 ± 51
CONV (*n* = 24)	14.8 ± 0.9	22.5 ± 0.6	20.5 ± 0.7	20.7 ± 0.5	1638 ± 133	79 ± 6	9.7 ± 1.0	372 ± 34	392 ± 36
**Variety (Va)**									
Kotsifali (*n* = 18)	14.6 ± 1.3	23.5 ± 0.5	20.7 ± 0.6	21.6 ± 0.6	1906 ± 131 **a**	114 ± 2 **a**	12.9 ± 1.2 **a**	356 ± 29 **a**	376 ± 31 **a**
Villana (*n* = 16)	15.7 ± 7.0	21.1 ± 0.7	19.3 ± 0.8	19.2 ± 0.7	1222 ± 113 **b**	57 ± 1 **b**	6.7 ± 0.8 **b**	9 ± 2 **b**	10 ± 2 **b**
Vidiano (*n* = 14)	12.5 ± 1.0	21.3 ± 0.6	19.4 ± 0.8	19.6 ± 0.6	1996 ± 192 **a**	59 ± 1 **b**	11.7 ± 1.6 **a**	21 ± 4 **b**	23 ± 4 **b**
**ANOVA results**									
(*p*-values)									
***Main effects***									
Yr	NS	**0.0201**	**0.0028**	**0.0078**	**0.0022**	**<0.0001**	**<0.0001**	*T*	*T*
PS	NS	NS	*T*	NS	NS	NS	0.0456	NS	NS
Va	NS	*T*	NS	*T*	**0.0014**	**<0.0001**	**0.0001**	**<0.0001**	**<0.0001**
***Interactions***									
Yr × PS	NS	NS	NS	NS	NS	NS	NS	NS	NS
Yr × Va	NS	NS	NS	NS	NS	**0.0067** ^**2**^	NS	**0.0275** ^**2**^	**0.0275** ^**2**^
PS × Va	NS	NS	NS	NS	NS	NS	**0.0160** ^**1**^	NS	NS
Yr × PS × Va	NS	NS	NS	NS	NS	NS	NS	NS	NS

GAE, gallic acid equivalents; The values presented are means ± standard error (SE); means with the same letter within the same column are not significantly different according to Tukey’s honestly significant difference test (*p* < 0.05); NS, not significant; ND, not determined: *T*, trend (0.1 > *p* > 0.05); **^1^** see Table 2 for interaction means (±SE); **^2^** see Table S2 (Supplementary Materials) for interaction means (±SE).

**Table 2:** Interactions means ± SE for the effects of variety farming system on total antioxidant activity in table grapes.

	Factor 1	Factor 2	
		Farming System	
Parameter	Variety	Organic	Conventional
Antioxidant activity (TEAC) µmol TE·g^−1^	Kotsifali	12.6 ± 1.7 **a A**	13.1 ± 1.7 **a A**
	Villana	7.1 ± 1.1 **b B**	6.4 ± 1.2 **b B**
	Vidiano	14.3 ± 2.4 **a A**	9.1 ± 1.6 **b B**

TE, Trolox equivalent; means labeled with the same lowercase letter within the same row and capital letters within the same column are not significant different (general linear hypothesis test *p* < 0.05).

**Table 3:** Antioxidant activity/capacity (TEAC and DPPH assays), total phenolic content (TPC), and total anthocyanin content (TAC) reported for different white grape varieties grown in organic and conventional production systems in different countries/regions.

Parameter Assessed.	Production System			Country	
*Grape Type* Grape Variety	Organic	Conventional	ANOVA Results	or Region (Study Type)	Reference
**TAA_TEAC_** (mM TE·L^−1^)					
Villana	7 ± 1.1	6 ± 1.2	NS	GR (FS)	this study
Vidiano	**14 ± 2.4**	9 ± 1.6	*	GR (FS)	this study
Early Sweet	5 ± 1.0	8 ± 1.4	NS	SA (RS)	[15] ^1^
Prime	6 ± 0.3	5 ± 0.5	NS	SA (RS)	[15] ^1^
Sugarlone	7 ± 1.3	4 ± 0.3	NS	SA (RS)	[15] ^1^
Thompson	6 ± 1.1	5 ± 0.6	NS	SA (RS)	[15] ^1^
**TAA_DPPH_** (mM TE·L^−1^)					
Villana	59 ± 1.7	58 ± 1.4	NS	GR (FS)	this study
Vidiano	57 ± 1.4	57 ± 1.4	NS	GR (FS)	this study
Early Sweet	54 ± 2.7	42 ± 0.4	NS	SA (RS)	[15] ^1^
Prime	53 ± 2.6	57 ± 2.0	NS	SA (RS)	[15] ^1^
Sugarlone	61 ± 1.6	60 ± 1.2	NS	SA (RS)	[15] ^1^
Thompson	61 ± 1.7	56 ± 1.8	NS	SA (RS)	[15] ^1^
Pignoletto	7 ± 0.8	8 ± 0.3	NS	IT (FS)	[22]
Albana	6 ± 0.1	7 ± 0.4	*	IT (FS)	[34]
Muscat Ottonel	16 ± 0.4	14 ± 0.2	NS	RO (FS)	[37]
Aromat de Iaşi	**3 ± 0.2**	2 ± 0.6	*	RO (FS)	[37]
Traminer roz	**7 ± 0.3**	5 ± 0.2	*	RO (FS)	[37]
Riesling italian	**9 ± 0.7**	8 ± 0.4	*	RO (FS)	[37]
Feteasca regală	10 ± 0.2	**12 ± 0.9**	*	RO (FS)	[37]
Timpuriu de Cluj	12 ± 1.1	**16 ± 1.9**	*	RO (FS)	[37]
**TPC** (mg GAE·kg^−1^)					
Villana	1201 ± 146	243 ± 182	NS	GR (FS)	this study
Vidiano	2243 ± 250	1748 ± 194	NS	GR (FS)	this study
Early Sweet	1180 ± 19	1328 ± 142	NS	SA (RS)	[15] ^1^
Prime	1088 ± 72	**1388 ± 35**	*	SA (RS)	[15] ^1^
Sugarlone	**1845 ± 87**	1556 ± 109	*	SA (RS)	[15] ^1^
Thompson	943 ± 113	861 ± 49	NS	SA (RS)	[15] ^1^
Muscat Ottonel	**631 ± 21**	41 ± 32	*	RO (FS)	[37]
Aromat de Iaşi	220 ± 14	228 ± 6	NS	RO (FS)	[37]
Traminer roz	219 ± 4	330 ± 2	NS	RO (FS)	[37]
Riesling italian	423 ± 10	436 ± 11	NS	RO (FS)	[37]
Feteasca regală	579 ± 11	575 ± 9	NS	RO (FS)	[37]
Timpuriu de Cluj	331 ± 4	380 ± 23	NS	RO (FS)	[37]
Niagara	**22 ± 1**	7 ± 1	*	BR (RS)	[38]
Niagara	**524 ± 7**	339 ± 7	*	BR (RS)	[13]

Means ± SE for the production system which resulted in significantly higher values are shown in **bold**. ^1^ Grape retail survey carried out in 2015. Study type: FS, farm survey; RS, retail survey. **TAA_T__EAC_/TAA_DPPH_,** total antioxidant activity/capacity; **TPC,** total phenolic concentration; **TAC,** total anthocyanin concentration. Asterisks within the same row denote that the mean for organic grapes is significantly different from the mean for conventional grapes (*p* < 0.05); NS within the same row denotes that the mean for organic grape is not significantly different from the mean for conventional grapes (*p* > 0.05). **GR**, Greece; **BR,** Brazil; **TR,** Turkey; **RO**, Romania; **FR**, France; **IT**, Italy; **SA**, South Africa; **MED**, Mediterranean countries.

**Table 4:** Total antioxidant activity/capacity reported for different red/black grape varieties grown in organic and conventional production systems in different countries/regions.

Parameter Assessed	Production System			Country	
*Grape Type* Grape Variety	Organic	Conventional	ANOVA Results	or Region (Study Type)	Reference
**TAA_TEAC_** (mM TE·L^−1^)					
Kotsifali	13 ± 1.7	13 ± 1.7	NS	GR (FS)	this study
Allison	5 ± 0.4	4 ± 0.1	NS	SA (RS)	[15] ^1^
Crimson	9 ± 1.6	5 ± 0.6	NS	SA (RS)	[15] ^1^
Flame	3 ± 0.2	2 ± 0.3	NS	SA (RS)	[15] ^1^
Sweet Celebration	12 ± 2.0	15 ± 0.9	NS	SA (RS)	[15] ^1^
Allison	11 ± 0.1	7 ± 2.6	NS	MED (RS)	[15] ^2^
Crimson	9 ± 1.3	9 ± 0.9	NS	MED (RS)	[15] ^1^
Flame	14 ± 3.4	16 ± 4.9	NS	MED (RS)	[15] ^2^
Scarlotta	6 ± 0.8	5 ± 1.0	NS	MED (RS)	[15] ^1^
Autumn Royal	16 ± 3.2	13 ± 1.5	NS	MED (RS)	[15] ^1^
Midnight Beauty	**30 ± 3.9**	17 ± 2.6	*	MED (RS)	[15] ^1^
Allison	7 ± 0.7	6 ± 0.4	NS	MED (RS)	[15] ^2^
Crimson	5 ± 0.8	6 ± 0.6	NS	MED (RS)	[15] ^2^
Bordo + Isabel	**52 ± 0.3**	31 ± 0.2	*	BR (RS)	[39]
Bordo	131 ± 1.7	131 ± 1.7	NS	BR (FS)	[7]
**TAA_DPPH_** (mM TE·L^−1^)					
Kotsifali	113 ± 2.7	115 ± 2.7	NS	GR (FS)	this study
Sangioves	25 ± 0.6	**31 ± 3.2**	*	IT (FS)	[22]
Allison	100 ± 3.4	102 ± 0.1	NS	SA (RS)	[15] ^1^
Crimson	97 ± 2.0	96 ± 1.5	NS	SA (RS)	[15] ^1^
Flame	51 ± 0.6	52 ± 2.4	NS	SA (RS)	[15] ^1^
Sweet Celebration	109 ± 6.4	108 ± 1.3	NS	SA (RS)	[15] ^1^
Allison	97 ± 3.5	102 ± 12.0	NS	MED (RS)	[15] ^2^
Crimson	103 ± 4.9	106 ± 6.4	NS	MED (RS)	[15] ^1^
Flame	157 ± 19.5	144 ± 28.3	NS	MED (RS)	[15] ^2^
Scarlotta	98 ± 4.4	90 ± 8.1	NS	MED (RS)	[15] ^1^
Autumn Royal	114 ± 8.3	105 ± 7.4	NS	MED (RS)	[15] ^1^
Midnight Beauty	**160 ± 12.9**	123 ± 14.2	*	MED (RS)	[15] ^1^
Allison	144 ± 2.7	142 ± 3.3	NS	MED (RS)	[15] ^2^
Crimson	141 ± 4.2	140 ± 1.6	NS	MED (RS)	[15] ^2^
Lambrusco	**26 ± 1.0**	20 ± 1.0	*	IT (FS)	[22]
Bordo + Isabel	**54 ± 0.2**	41 ± 0.7	*	BR (RS)	[39]
Bordo	77 ± 3.4	102 ± 1.7	NS	BR (FS)	[7]
Bordo	**146 ± 1**	126 ± 2	*	BR (RS)	[40]
Napoca	32 ± 1.4	25 ± 1.3	NS	RO (FS)	[37]
Muscat Hamburg	23 ± 0.1	23 ± 0.5	NS	RO (FS)	[37]

Means ± SE for the production system which resulted in significantly higher values are shown in **bold**. ^1^ Grape retail survey carried out in 2015; ^2^ grape retail survey carried out in 2012. Study type: FS, farm Survey; RS, retail survey. **TAA_T__EAC_/TAA_DPPH_**, total antioxidant activity/capacity. Asterisks within the same row denote that the mean for organic grapes is significantly different from the mean for conventional grapes (*p* < 0.05); NS within the same row denotes that the mean for organic grape is not significantly different from the mean for conventional grapes (*p* > 0.05). **GR**, Greece; **BR**, Brazil; **TR**, Turkey; **RO**, Romania; **FR**, France; **IT**, Italy; **SA**, South Africa; **MED**, Mediterranean countries.

**Table 5:** Total phenolic content (TPC) and total anthocyanin content (TAC) reported for different red/black grape varieties grown in organic and conventional production systems in different countries.

Parameter Assessed	Production System			Country	
*Grape Type* Grape Variety	Organic	Conventional	ANOVA Results	or Region (Study Type)	Reference
**TPC (mg GAE·kg^−1^)**					
Kotsifali	1938 ± 187	1903 ± 94	NS	GR (FS)	this study
Allison	1838 ± 83	1866 ± 87	NS	SA (RS)	[15] ^1^
Crimson	1416 ± 101	1296 ± 47	NS	SA (RS)	[15] ^1^
Flame	2083 ± 299	1784 ± 243	NS	SA (RS)	[15] ^1^
Sweet Celebration	1824 ± 104	1804 ± 219	NS	SA (RS)	[15] ^1^
Allison	1768 ± 676	2058 ± 29	NS	MED (RS)	[15] ^1^
Crimson	2012 ± 113	1876 ± 109	NS	MED (RS)	[15] ^1^
Flame	2769 ± 462	2511 ± 347	NS	MED (RS)	[15]^2^
Scarlotta	**2159 ± 292**	1494 ± 419	*	MED (RS)	[15] ^1^
Autumn Royal	2213 ± 559	1925 ± 535	NS	MED (RS)	[15] ^1^
Midnight Beauty	3173 ± 261	2435 ± 108	NS	MED (RS)	[15] ^1^
Allison	2154 ± 230	1914 ± 41	NS	MED (RS)	[15] ^2^
Crimson	1942 ± 188	2356 ± 178	NS	MED (RS)	[15] ^2^
Bord±sabel	**3378 ± 50**	2015 ± 22	*	BR (RS)	[44]
Bordo	2724 ± 56	**3636 ± 72**	*	BR (FS)	[7]
Bordo	**3346 ± 17**	1985 ± 56	*	BR (RS)	[13]
Bordo	**146 ± 1**	126 ± 2	*	BR (RS)	[40]
Napoca	1341 ± 21	1231 ± 21	NS	RO (FS)	[37]
Muscat Hamburg	978 ± 13	953 ± 10	NS	RO (FS)	[37]
**TAC** (mg cyan·L^−1^)					
Kotsifali	341 ± 48	372 ± 34	NS	GR (FS)	this study
Allison	109 ± 35	174 ± 15	NS	SA (RS)	[15] ^1^
Crimson	72 ± 12	131 ± 16	NS	SA (RS)	[15] ^1^
Flame	128 ± 28	75 ± 16	NS	SA (RS)	[15] ^1^
Sweet Celebration	97 ± 13	94 ± 8	NS	SA (RS)	[15] ^1^
Allison	49 ± 5	118 ± 58	NS	MED (RS)	[15] ^1^
Crimson	67 ± 12	91 ± 16	NS	MED (RS)	[15] ^1^
Flame	93 ± 14	77 ± 11	NS	MED (RS)	[15] ^2^
Scarlotta	43 ± 10	**196 ± 139**	*	MED (RS)	[15] ^1^
Autumn Royal	177 ± 36	97 ± 99	NS	MED (RS)	[15] ^1^
Midnight Beauty	**851 ± 110**	499 ± 64	*	MED (RS)	[15] ^1^
Allison	**74 ± 21**	208 ± 67	NS	MED (RS)	[15] ^2^
Crimson	81 ± 4	161 ± 58	NS	MED (RS)	[15] ^2^
Bordo	**341 ± 1**	255 ± 1	*	BR (RS)	[40]
Monastrell	**721 ± 35**	518 ± 26	*	SP (FS)	[1]
Syrah	897	1277	NS	FR (EX)	[2]

Means ± SE for the production system which resulted in significantly higher values are shown in **bold**. ^1^ Grape retail survey carried out in 2015; ^2^ grape retail survey carried out in 2016. Study type: EX, field experiment; FS, farm survey; RS, retail survey. **TPC,** total phenolic concentration; **TAC,** total anthocyanin concentration. Asterisks within the same row denote that the mean for organic grapes is significantly different from the mean for conventional grapes (*p* < 0.05); NS within the same row denotes that the mean for organic grape is not significantly different from the mean for conventional grapes (*p* > 0.05). **GR**, Greece; **BR,** Brazil; **TR,** Turkey; **RO**, Romania; **FR**, France; **IT**, Italy; **SA**, South Africa; **MED**, Mediterranean countries.

## Data Availability

Data will be made available upon reasonable request by author Gultakin Hasanaliyeva.

## Supplementary Materials

The following are available online at https://www.mdpi.com/2304-8158/10/2/476/s1: Table S1. Farm/orchard characteristics and agronomic parameters used for redundancy analyses (RDA); Table S2. Interaction means ± SE for the effects of variety and year/ production season on total antioxidant activity (DPPH) and anthocyanin concentrations in table grapes; Table S3. Effect of, and interaction between, production system and year on concentrations of individual anthocyanins (*delphinidin-3-O-glucoside, cyanidin-3-O-glucoside, petunidin-3-O-glucoside, peonidin-3-O-glucoside, malvidin-3-O-glucoside, peonidin-3-O-(6”-p-coumaroyl) glucoside, malvidin-3-O-(6”p-coumaroyl)glucoside*) in table grapes of the red variety Kotsifali (two-factor ANOVA); Figure S1. Biplot derived from the redundancy analysis showing the relationship between variety, agronomic, and orchard site and soil explanatory variables/drivers and grape yield and quality parameters.
