# Supplementary material for: Effect of Organic and Conventional Production Methods on Fruit Yield and Nutritional Quality Parameters in Three Traditional Cretan Grape Varieties: Results from a Farm Survey
Source: Foods. 2021 Feb 22;10(2):476. doi: 10.3390/foods10020476 (PMC7926479; doi:10.3390/foods10020476)
Supplement: Supplementary file 1 [file foods-10-00476-s001.pdf]

Supplementary Materials

Table S1. Farm/orchard characteristics and agronomic parameters used for redundancy analyses (RDA; see Figures 1 and S1). Data were obtained via structured questionnaire based farmer surveys.

**Farm**	**Farm Type**	**Grape Variety**	**Vineyard Age** years	**Soil Texture**	**Orchard Orientation***
1	Conventional	Kotsifali	18	clay loam	west
2	Conventional	Kotsifali	20	clay loam	west
3	Conventional	Kotsifali	16	clay loam	south
4	Conventional	Kotsifali	13	clay loam	south east
5	Conventional	Kotsifali	20	clay loam	west
6(a)	Organic	Kotsifali	21	clay loam	west
7(b)	Organic	Kotsifali	22	clay loam	west
8	Organic	Kotsifali	22	sandy loam	south
9	Organic	Kotsifali	17	clay loam	west
10	Organic	Kotsifali	7	clay loam	south
11	Conventional	Vidiano	7	clay loam	south
12	Conventional	Vidiano	4	clay loam	south
13	Conventional	Vidiano	16	clay loam	south
14	Conventional	Vidiano	5	clay loam	west
15	Organic	Vidiano	4	clay loam	west
16	Organic	Vidiano	10	clay loam	south
17	Organic	Vidiano	3	sandy loam	south
18	Organic	Vidiano	25	clay loam	south
19(a)	Conventional	Villana	18	clay loam	west
20(b)	Conventional	Villana	19	clay loam	south
21	Conventional	Villana	20	clay loam	west
22	Conventional	Villana	13	clay loam	south
23	Organic	Villana	21	clay loam	west
24	Organic	Villana	14	clay loam	west
25	Organic	Villana	20	clay loam	west
26	Organic	Villana	20	clay loam	west

* direction the slope of the orchard is facing

Table S1 cont.. Farm/orchard characteristics and agronomic parameters used for redundancy analyses (RDA; see Figures 1 and S1). Data were obtained via structured questionnaire based farmer surveys.

				Estimated total N, P K inputs from fertilisers			
**Farm**	**Farm Type**	**Irrigation**	**Plant Density**	**N** kg ha^{-1} $year^{-1}$	**K** kg ha^{-1} $year^{-1}$	**P** kg ha^{-1} $year^{-1}$	**Type of fertiliser used**
1	Conventional	dripping	300	110	150	150	mineral
2	Conventional	no	300	33	40	40	mineral
3	Conventional	dripping	300	4.3	13	0.109	leonardite
4	Conventional	no	220	110	150	150	mineral
5	Conventional	no	400	400	160	0	mineral
6(a)	Organic	dripping	300	50	100	50	sheep manure
7(b)	Organic	no	350	50	100	50	sheep manure
8	Organic	no	220	84	108	36	sheep manure
9	Organic	no	350	45	15	60	grape waste compost
10	Organic	dripping	300	70	90	30	sheep manure
11	Conventional	no	300	33	40	40	mineral
12	Conventional	dripping	350	150	160	0	mineral
13	Conventional	dripping	320	4.3	13	0.109	leonardite
14	Conventional	no	300	110	150	150	mineral
15	Organic	no	360	160	160	0	mineral
16	Organic	no	450	45	15	60	grape waste compost
17	Organic	dripping	260	0	0	0	mineral
18	Organic	no	350	70	90	30	sheep manure
19(a)	Conventional	dripping	300	110	150	150	mineral
20(b)	Conventional	no	300	110	150	150	mineral
21	Conventional	no	300	33	40	40	mineral
22	Conventional	dripping	320	4.3	13	0.109	leonardite
23	Organic	dripping	300	50	100	50	sheep manure
24	Organic	no	220	84	108	36	sheep manure
25	Organic	no	280	70	90	30	sheep manure
26	Organic	no	350	45	15	60	grape waste compost

Table S2. Number of wine samples* produced in different years (vintages) collected and analysed

Year/vintage**	Red wine made from Kotsifali grapes		White wine made from Vidiano grapes	
	**Organic**	**Conventional**	**Organic**	**Conventional**
2009	1	1		
2010	1	1		
2011	2	2	1	
2012	4	4	2	1
2013	2	2	2	2
2014		1	5	5
**Total number of samples analysed**	**10**	**11**	**10**	**8**

*, a sample constitute a bottle or box (3 l or 5 l) of wine; ** if more than one sample was collected from a specific production year/vintage each sample was from a different winery.

Table S3. Interactions means ± SE for the effects of variety and year/ production season on total antioxidant activity (DPPH) and anthocyanin concentrations in table grapes

**Parameter assessed**	**Factor 1 Variety**	**Factor 2 Year/ production season**	
		**2014**	**2015**
**Antioxidant activity (DPPH)** μmol TE g^{-1}	Kotsifali (red)	122 ±1.0 **a A**	108 ±1.1 **b A**
	Villana (white)	63 ±0.6 **a B**	55 ±0.4 **b B**
	Vidiano (white	61 ±0.2 **a B**	54 ±0.4 **b B**
**Anthocyanin concentrations** mg cyan/ kg^{-1}	Kotsifali (red)	456 ±50 **a A**	313 ±25 **b A**
	Villana (white)	8 ± 4 **a B**	12 ± 2 **a B**
	Vidiano (white	17 ± 6 **a B**	27 ± 6 **a B**

For each parameter's assessed means labelled with the same lower case letter within the same row and capital letters within the same column are not significant different (General Linear Hypothesis test p<0.05)

Table S4. Effect of, and interaction between, production system and year on concentrations of individual anthocyanins (d*elphinidin-3-O-glucoside, cyanidin-3-O-glucoside, petunidin-3-O-glucoside, peonidin-3-O-glucoside, malvidin-3-O-glucoside, peonidin-3-O-(6″-p-coumaroyl)glucoside, malvidin-3-O(6″-p-coumaroyl)glucoside* in table grapes of the red variety Kotsifali (2-factor ANOVA)

Factor	Delphinidin 3-O-glucoside	Cyanidin 3-O-glucoside	Petunidin 3-O-glucoside	Peonidin 3-O-glucoside	Malvidin 3-O-glucoside	Peonidin 3-O-p-coumaroyl glucoside	Malvidin 3-O-p-coumaroyl-glucoside
**Year (Yr)**							
2014 (*n*=8)	6.4 ± 1.7	15.9 ± 3.8	11.6 ± 2.3	87 ± 13	82 ± 10	6.3 ± 1.1	7.6 ± 2.1
2015 (*n*=10)	3.1 ± 0.6	12.0 ± 3.4	7.4 ± 0.9	84 ± 18	66 ± 5	4.6 ± 1.6	6.4 ± 4.1
**Production system (PS)**							
ORG (*n*=9)	4.1 ± 1.3	14.9 ± 4.2	8.9 ± 1.7	95 ± 19	72 ± 8	4.5 ± 1.0	4.5 ± 2.0
CONV (*n*=9)	4.9 ± 1.3	12.6 ± 2.9	9.6 ± 1.7	76 ± 14	74 ± 7	6.3 ± 1.8	9.4 ± 4.4
**ANOVA** (*P*-values)							
Main effects							
Yr	NS	NS	NS	NS	NS	NS	NS
PS	NS	NS	NS	NS	NS	NS	NS
Interaction							
Yr : PS	NS	NS	NS	NS	NS	NS	NS

The values presented as means±SE; Mean values are expressed as mg kg^{-1} FW; NS, not significant

Table S5. Effect of, and interaction between, production system (organic [ORG] vs conventional [CONV]), and variety for the total phenolic content (TPC), total antioxidant activity (TAA) by DPPH /TEAC assays and total anthocyanin content (TAC) (expressed as cyanidin-3-glucoside [cyan] or malvidin-3-glucoside [mal] equivalents) in Kotsifali (red) and Vidiano (white) wine samples (2-factor ANOVA for TPC and TAA; 1-factor ANOVA for TAC)

**Factors**	TPC mg GAE l^{-1}	TAA (DPPH) mM TE l^{-1}	TAA (TEAC) mM TE l^{-1}	TAC mg cyan l^{-1}	TAC mg mal l^{-1}
**Production system (PS)**					
ORG (*n*=20)	1486 ±211	5.2 ±0.7	4.3 ±0.2	66 ±16	69 ±17
CONV (*n*=20)	1478 ±224	5.1 ±0.7	4.1 ±0.3	109 ±11	115 ±12
**Main grape variety (Va) used for wine-making**					
Kotsifali (100%) (*n*=12)	2279 ± 70 **a**	7.8 ±0.2 **a**	5.2 ±0.002 **a**	92 ±16	97 ±17
Kotsifali (70%) (*n*=10)	2333 ±159 **a**	8.0 ±0.5 **a**	5.2 ±0.002 **a**	83 ±14	87 ±14
Vidiano (100%) (*n*=14)	504 ± 26 **b**	1.9 ±0.3 **b**	3.2 ±0.20 **b**	ND	ND
Vidiano (70%) (*n*=4)	389 ± 7 **b**	1.6 ±0.5 **b**	2.8 ±0.33 **b**		
**ANOVA** (*P*-values)					
Main effects					
PS	NS	NS	NS	**0.036**	**0.036**
Va	**<0.0001**	**<0.0001**	**0.0001**		
Interaction					
PS : Va	NS	NS	NS		

The values presented as means ±SE; means with same lower case letter within the same column are not significant different (General Linear Hypothesis test; $p<0.05$); NS, not significant; ND, not determined.

Table S6. Effect of production system (organic [ORG] vs conventional [CONV]) on concentrations of individual anthocyanins (d*elphinidin-3-O-glucoside, cyanidin-3-O-glucoside, petunidin-3-O-glucoside, peonidin-3-O-glucoside, malvidin-3-O-glucoside, peonidin-3-O-(6″-p-coumaroyl)glucoside, malvidin-3-O-(6″-p-coumaroyl)glucoside)* in wines made from grapes of the red variety Kotsifali (1-factor ANOVA)

**Factor**	Delphinidin 3-O-glucoside	Cyanidin 3-O-glucoside	Petunidin 3-O-glucoside	Peonidin 3-O-glucoside	Malvidin 3-O-glucoside	Malvidin 3-O -p-coumaroylglucoside
**Production system (PS)**						
ORG (*n*=7)	1.02 ±0.55	0.21 ±0.12	1.13 ±0.67	1.36 ±0.74	7.01 ±4.15	0.58 ±0.36
CONV (*n*=6)	1.00 ±0.23	0.25 ±0.04	1.31 ±0.32	1.92 ±0.47	10.88 ±3.15	0.81 ±0.26
**ANOVA p values**						
PS	NS	NS	NS	NS	NS	NS

The values presented as means ±SE; Mean values are expressed as mg kg^{-1} FW; NS, not significant

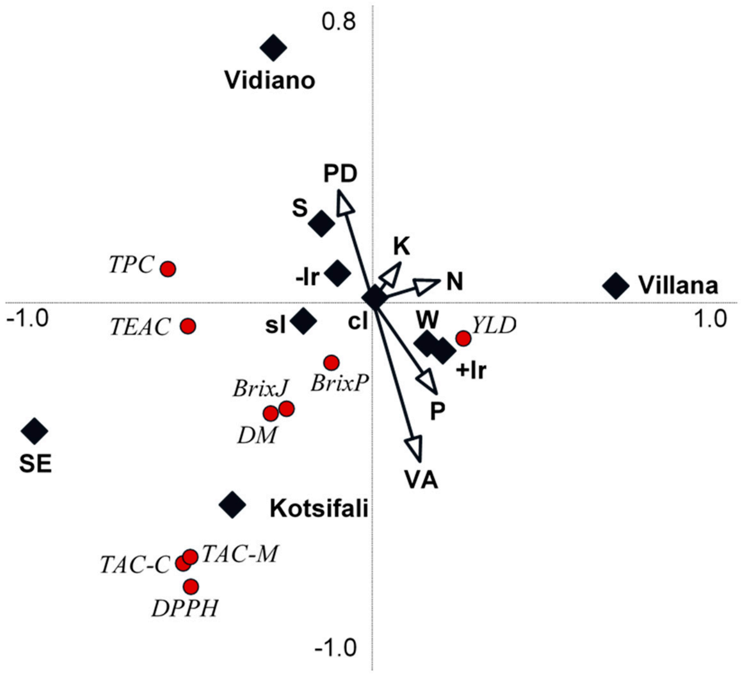

Response variables (●)
YLD, grape yield
TPC, phenolic content
TEAC, antioxidant activity (ABTS assay)
DPPH, antioxidant activity (DPPH)
BrixP, sugar content in pulp
BrixJ, sugar content in juice
DM, grape dry matter content
TAC-M, anthocyanin content (cyanidin-3-O-glucoside equivalents)
TAC-C, anthocyanin content (malvidin-3-O-glucoside equivalents)

Continuous explanatory variables (—▷)
VA, vineyard age (*F*-value=0.3; *P*=0.62)
PD, plant density (*F*<0.1; *P*=0.9),
N, total N-input* (*F*=0.3; *P*=0.61)
K, total K-input* (*F*=0.3; *P*=0.61)
P, total P-input* (*F*=0.6; *P*=0.5)

Figure 2. Biplot derived from the redundancy analysis showing the relationship between variety, agronomic, and orchard site and soil explanatory variables/drivers and grape yield and quality parameters.
Eigenvalues were 32.5% and 8.6% for Axis 1 and 2 respectively.
Fixed explanatory variables (◆):
(a) variety: **Vil,** Villana (*F*=17.1, *P*=0.002); **Vid,** Vidiano (*F*=5.7, *P*=0.024); **Kot,** Kotsifali (*F*=5.7; *P*=0.024)
(b) irrigation: **+Ir** , with drip irrigation (*F*=1.3, *P*=0.31); **-Ir**, without drip irrigation (*F*=1.3, *P*=0.31)
(c) orchard orientation, facing: **W,** west (*F*=0.9, *P*=0.36); **S,** south (*F*=1.0, *P*=0.31); **SE**, south east (*F*=1.0, *P*=0.31)
(d) soil texture: **cl,** clay loam (*F*=0.2, *P*=0.74); **sl,** sandy loam (*F*=0.2, *P*=0.74).
*, from mineral and/or organic fertilizer;
